# Marathon Race Affects Neutrophil Surface Molecules: Role of Inflammatory Mediators

**DOI:** 10.1371/journal.pone.0166687

**Published:** 2016-12-02

**Authors:** Vinicius Coneglian Santos, Ana Paula Renno Sierra, Rodrigo Oliveira, Kim Guimarães Caçula, César Miguel Momesso, Fabio Takeo Sato, Maysa Braga Barros Silva, Heloisa Helena Oliveira, Maria Elizabeth Pereira Passos, Diego Ribeiro de Souza, Olivia Santos Gondim, Marino Benetti, Adriana Cristina Levada-Pires, Nabil Ghorayeb, Maria Augusta Peduti Dal Molin Kiss, Renata Gorjão, Tânia Cristina Pithon-Curi, Maria Fernanda Cury-Boaventura

**Affiliations:** 1 Institute of Biomedical Sciences, University of São Paulo, São Paulo, São Paulo, Brazil; 2 Institute of Physical Activity and Sports Sciences, University of Cruzeiro do Sul, São Paulo, São Paulo, Brazil; 3 School of Physical Education and Sport, University of São Paulo, São Paulo, São Paulo, Brazil; 4 Sports Cardiology Department, Dante Pazzanese Institute of Cardiology, São Paulo, São Paulo, Brazil; 5 Medicine Department, Nove de Julho University, São Paulo, São Paulo, Brazil; Universitat de Valencia, SPAIN

## Abstract

The fatigue induced by marathon races was observed in terms of inflammatory and immunological outcomes. Neutrophil survival and activation are essential for inflammation resolution and contributes directly to the pathogenesis of many infectious and inflammatory conditions. The aim of this study was to investigate the effect of marathon races on surface molecules related to neutrophil adhesion and extrinsic apoptosis pathway and its association with inflammatory markers. We evaluated 23 trained male runners at the São Paulo International Marathon 2013. The following components were measured: hematological and inflammatory mediators, muscle damage markers, and neutrophil function. The marathon race induced an increased leukocyte and neutrophil counts; creatine kinase (CK), lactate dehydrogenase (LDH), CK-MB, interleukin (IL)-6, IL-10, and IL-8 levels. C-reactive protein (CRP), IL-12, and tumor necrosis factor (TNF)-α plasma concentrations were significantly higher 24 h and 72 h after the marathon race. Hemoglobin and hematocrit levels decreased 72 h after the marathon race. We also observed an increased intercellular adhesion molecule-1 (ICAM-1) expression and decreasedTNF receptor-1 (TNFR1) expression immediately after and 24 h after the marathon race. We observed an increased DNA fragmentation and L-selectin and Fas receptor expressions in the recovery period, indicating a possible slow rolling phase and delayed neutrophil activation and apoptosis. Marathon racing affects neutrophils adhesion and survival in the course of inflammation, supporting the “open-window” post-exercise hypothesis.

## Introduction

Marathon races have been increasing in the last few years. The fatigue induced by marathon races has been observed on inflammation and immunological consequences [1, 2]. Marathon runners have been demonstrated to have an acute inflammatory response during the race, involving inflammatory mediators. Interleukin (IL)-6 is the first cytokine to appear in circulation during exercise, followed by anti-inflammatory cytokines such as IL-1ra, IL-4, and IL-10. IL-4 and IL-10 prevent inflammatory tissue damage but also cause immunosuppressive states after exercise [3–5]. High levels of strenuous exercise decrease both innate and acquired immunity, typically 15–25%, however, remains unclear the role of changes in immunity on upper respiratory tract infection (URTI) susceptibility [1]. The prevalence of upper respiratory tract symptoms (URTS) was 50% in runners of the London marathon race [6]; severity of URTs at 24 h and 48 h following the marathon race was also significantly greater than that at baseline [7].

Cytokines are potent functional modulators of cellular immunity altering neutrophil function [8–10]. Neutrophils play an important role in clearing exercise-induced tissue damage, inflammatory effectors, and immunoregulatory cells [11, 12]. Neutrophilia has been associated with hypoxia, and neutropenia markedly impairs innate defense and increases susceptibility to infection. During and after exercise one of the more pronounced features of endurance exercise on immune response is prolonged neutrophilia and impaired neutrophil function [10, 13, 14]. Previous study suggested that neutrophil activation and infiltration follows exercise-induced muscle injury [15]. Under inflammatory conditions, neutrophil recruitment is initiated by selectin family molecules that facilitate cell rolling, followed by chemotactic activation of integrins that interact with ICAM, leading to transendothelial migration [16]. After extravasation, precise regulation of neutrophil survival is essential for the resolution of inflammation and contributes directly to the pathogenesis of many infectious and inflammatory disease states [17]. Neutrophil apoptosis can be triggered via receptor death (extrinsic pathway) or mitochondria-mediated pathway (intrinsic pathway). The extrinsic pathway appears to play a more important role in neutrophil turnover during infection and inflammation [17].

The expression of surface molecules related to neutrophil trafficking and extrinsic apoptosis pathway and the association with inflammatory and damage markers remains unclear. The aim of this study was to determine whether running a marathon race affects neutrophil function and to characterize the underlying mechanisms.

## Methods

### Subjects

Twenty-three Brazilian male endurance runners participated in this study. Measurements of total body mass (kg), height (cm), Body Mass Index (BMI, kg/m^2^) and body fat (%) were conducted according to the International Society for the Advancement of Kinanthropometry and expressed as the mean ± SEM. The demographic data for these subjects are summarized as follows: age, 34 ± 6 years; height, 173 ± 0 cm; body mass, 75 ± 6 kg; % of fat mass, 18 ± 4; body mass index, 25 ± 2; race duration, 257 ± 34 minutes; oxygen consumption peak (VO_2peak_), 48.7 ± 5 mL.kg^-1^.min^-1^. An advertisement was placed on the São Paulo International Marathon 2013 website and an email was sent to all runners to recruit participants. The participants were not using medications at the time of blood collections. The study protocol was approved by the Ethics Committee of the University of São Paulo, São Paulo, Brazil (Permit Number: 979/2010), experimental procedures were performed in accordance with the Declaration of Helsinki and all individuals signed an informed consent form.

### Experimental design

Anthropometrics parameters and cardiopulmonary exercise test were performed one to two weeks before marathon. We applied the Wisconsin Upper Respiratory Symptom Survey (WURSS-21) one day before marathon race and two weeks after marathon. Runners reported incidence of cough; colored discharge; sore throat; watery eyes; nasal symptoms (congestion and/or discharge); sneezing and rate their severity on a 7-point anchored by 1 (very mild) to 7 (very strong). The degree of functional limitation induced by upper respiratory symptoms such as was think clearly, speak clearly, sleep well, breathe easily and live your personal life also was evaluated using the same rate severity.

Functional capacity was assessed by means of cardiopulmonary exercise test (CEPT) with expired gas analysis, performed on a treadmill (TEB Apex 200, TEB, São Paulo, Brazil, speed 0-24km/h, grade 0%-35%) 7 to 15 days before marathon race. A protocol was used, with a starting speed of 8 km/h and grade of 1%; speed was then increased every 1 minute in 1 km/h. The objective was to achieve fatigue within 8 to 12 minutes. Blood pressure was measured with a sphygmomanometer in the beginning of the test. Respiratory gas analysis was performed by the K4b2 system (Cosmed, Rome, Italy) in breath by breath mode.

Tests were considered maximum when at least three of these features were attained: limiting symptoms/intense physical fatigue, increase in VO_2_ lower than 2.1 mL.kg^-1^.min^-1^ through an increase in the speed, attained maximal heart rate or respiratory quotient higher than 1.1.

Blood samples (30 mL) were collected in vacuum tubes containing an anticoagulant (0.004% EDTA) 24 h before, immediately after, 24 h after and 72 h the after São Paulo International Marathon 2013. Cellular and biochemical analyses were subsequently performed at Institute of Physical Activity Sciences and Sports of the Cruzeiro do Sul University and at Clinical Laboratory of Dante Pazzanese Institute of Cardiology. After centrifugation, plasma was aliquoted, frozen, and stored at -80°C for later analysis. Neutrophils were separated from whole blood of runners immediately after race.

The weather parameters at the São Paulo International Marathon 2013 between 8 am and 1pm were: average temperature 17.9°C, maximum temperature 20.2°C and minimum temperature 15.6°C, with a relative humidity of 60.7%, maximum relative humidity of 76% and minimum relative humidity of 54%.

### Hematological and inflammatory parameters

Hematological parameters (leukocyte and neutrophil count, hemoglobin and hematocrit) and C-reactive protein were performed in Clinical Laboratory of Dante Pazzanese Institute of Cardiology, with routine automated methodology (cytochemical/isovolumetric and kinetic assay, respectively) immediately after collection. The plasma levels of IL-6, IL-1β, IL-10, IL-8, IL-12p70 and TNF-α were determined using BD ^™^ Human Inflammatory Cytokine Citometric Bead Array Kit and BD Accuri cytometer according to manufacturer's instructions (BD Biosciences, San Jose, CA, USA).

### Muscle damage analyses

Muscle damage analyses were performed in Clinical Laboratory of Dante Pazzanese Institute of Cardiology, with routine automated methodology. Muscle damage markers were evaluated based on measurements of plasma CK, CK-MB, and LDH activities by kinetic-enzymatic method which was performed 24 hours after collection.

### Measurement of neutrophil function

Neutrophils were separated from whole blood of runners on Histopaque-1077 (d = 1.077) gradients (Sigma-Aldrich, St. Louis, MO, USA) (Boyum et al. 1968). The neutrophils obtained from the blood sample athletes were counted in a Neubauer chamber under an optical microscope (Nikon, Melville, NY, USA). Neutrophils (1x10^6^ cells / ml) were incubated with antibody conjugated to fluorescein isothiocyanate (FITC), phycoerythrin (PE) or allophycocyanin (APC) for 30 minutes in the dark at room temperature. The expression of surface molecules ICAM-1 (CD54—FITC), L-selectin (CD62—FITC), TNFR-1 receptor (CD120b—PE) and Fas (CD95 –APC) were analyzed by flow cytometry. The fluorescence was determined by flow cytometry at wavelengths 530/30 nm (FITC) 660/20 nm (APC) or 695/40 nm (PE) (BD Accuri cytometer) and ten thousand events per sample were acquired in histograms.

DNA fragmentation was analyzed by flow cytometry (BD Accuri cytometer) after DNA staining with propidium iodide, according to the method described by Nicoletti et al. (1991) [18].

### Statistical analyses

Statistical analyses were performed using Statistical Package for the Social Sciences (IBM SPSS Statistics for Mac, Version 22.0. Armonk, NY, USA). The normality of the data distribution was determined by the Kolmogorov-Smirnov test and reject the normality. Differences between the steps (before, immediately after, 24h after and 72h after the race) were tested for significance by repeated measures one way ANOVA with Geisser-Greenhouse correction and Tukey`s multiple comparison test. The difference values between the steps were calculated with the value post—value before. Spearman test was applied to analyze correlation between the variables. Statistical significance was assumed at a p-value <0.05.

The GraphPad Prism 5 software program (Graph Pad Software, Inc., San Diego, CA, USA) was used for the graphics.

## Results

The incidence of URTs was significantly greater than that at baseline from 13% to 26% after 1–2 weeks (p<0.05). The marathon runners reported an increase in the sum of severity of upper respiratory infection symptoms (from 27 to 71) and the sum of functional impairments (from 15 to 66) after the marathon race.

**Fig 1:**
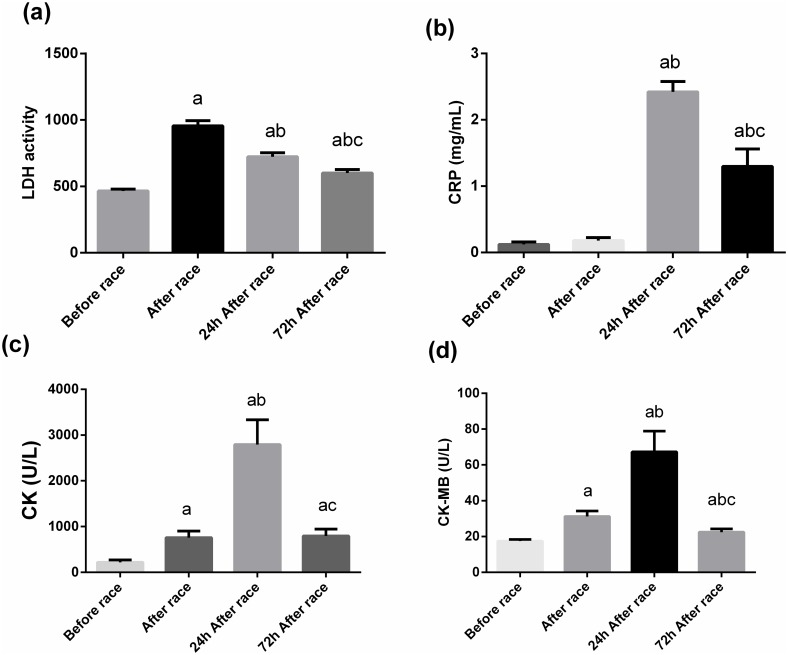
Effect of the marathon race on muscle damage markers and CRP. The plasma was separated before, immediately after, 24 h after, and 72 h after the marathon race. The plasma activities of LDH (A), CK (C), CK-MB (D), and plasma levels of CRP (B) were determined. The values presented are the mean ± SEM of 23 runners.^a^ p<0.001 vs before the marathon race, ^b^ p<0.0001 vs immediately after the marathon race, and ^c^ p<0.01 vs 24 h after the marathon race.

The marathon race induced an increased LDH, CK-MB and CK activities immediately after the marathon race (by 2-fold, 2-fold and 3.5-fold p<0.001), 24 h after (by 1.5-, 3.9-fold and 12.8-fold, respectively, p<0.0001) and 72 h after the marathon race (by 1.3-fold, 1.3-fold and 3.5-fold, respectively, p<0.01) (Fig 1A, 1C and 1D). C-reactive protein (CRP) levels increased 24 h after the competition (by 20-fold) and remained high 72 h after the competition (by 10-fold, p<0.0001) (Fig 1B). The VO_2peak_ was negatively correlated with CK (−0.42, p<0.05) and LDH (−0.48, p<0.05) 24 h after competition. In the same way, race duration was positively correlated with LDH, CK, and CK-MB immediately after (r = 0.52, r = 0.48 and 0.57, respectively, p < 0.05) and 24 h after the race(r = 0.58, r = 0.52 and 0.72, respectively, p < 0.05).

**Fig 2:**
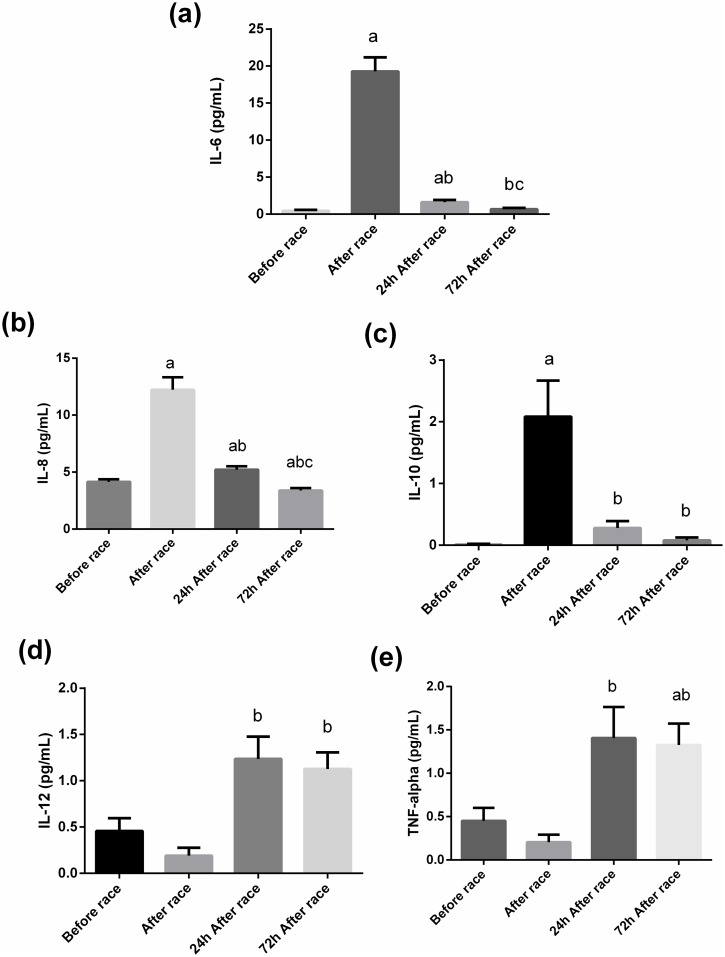
Effect of the marathon race on plasma cytokines. Plasma concentrations of IL-6 (A), IL-8 (B) IL-10 (C), IL-12 (D), and TNF-alpha (E) were determined using cytometric bead array. The values presented are the mean ± SEM of 23 runners.^a^p<0.05 vs before the marathon race, ^b^p<0.05vs immediately after the marathon race, and ^c^ p<0.05 vs 24 h after the marathon race.

IL-10, IL-6, and IL-8 plasma concentrations significantly increased immediately after the marathon race by 200-, 42-, and 10-fold, respectively (p<0.01). IL-10 and IL-6 levels returned to the baseline 24 h after the marathon race and IL-8 decreased 72 h after the marathon race (Fig 2A–2C). IL-12 and TNF-α plasma levels were significantly higher 24 and 72 h after the marathon race by approximately 5- to 7-fold compared with those immediately after the marathon race (p<0.05) (Fig 2D and 2E). There was no change in IL-1β plasma concentration (data not shown). TNF-α was positively correlated with IL-12 immediately after, 24 h after, and 72 h after the competition (r = 0.43, 0.68, and 0.49, respectively, p<0.05), with IL-6 24 and 72 h after the competition (r = 0.5 and 0.72, respectively, p<0.05), with IL-8 after the competition (r = 0.49, p<0.05), and with IL-10 24 h after the competition (r = 0.47, p<0.05).

**Fig 3:**
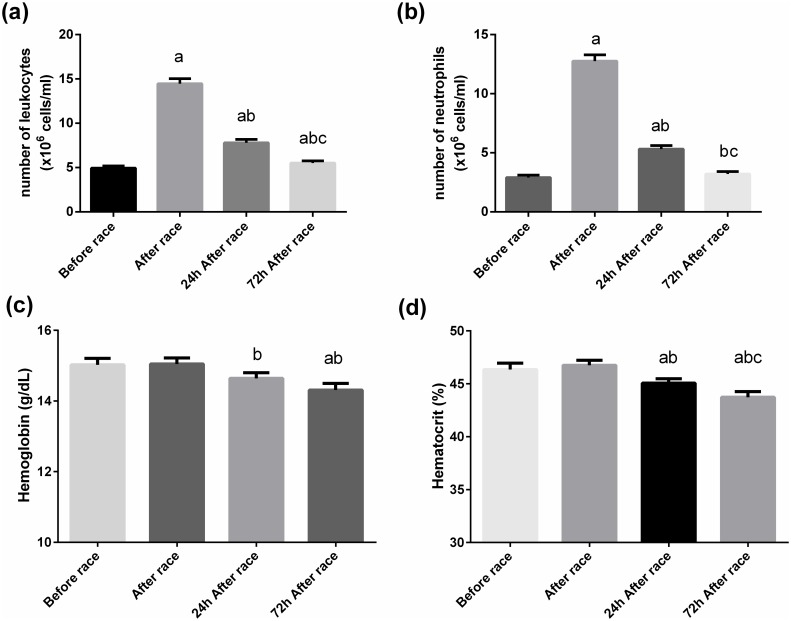
Effect of the marathon race on hematological parameters. Total leukocyte number (A), neutrophil number (B), hematocrit (C), and hemoglobin (D) were determined before, immediately after, 24 h, and 72 h after the marathon race. The values presented are the mean ± SEM of 23 runners.^a^p<0.05 vs before the marathon race, ^b^p<0.05 vs immediately after the marathon race, and ^c^p<0.05 vs 24 h after the marathon race.

The marathon race increased 4-fold the leukocytes (from 5.1 ± 1 to 14.3 ± 3 × 10^3^ μl) and neutrophils counts (from 3 ± 1 to 12 ± 3 × 10^3^ μl), and neutrophils returned to baseline levels 72 h after the marathon race (to 3 ± 1 × 10^3^ μl, respectively, p<0.0001) (Fig 3A and 3B). Hemoglobin levels decreased 72 h after the marathon race and Hematocrit levels were approximately 5% reduced at 24 h and 72 h after the marathon race(p<0.01) (Fig 3C and 3D). Immediately after the marathon race, leukocytes and neutrophils counts were negatively correlated with TNF-α levels (r = −0.44 and −0.47, respectively, p<0.05) and positively correlated with CK activity (r = 0.45 and 0.52, respectively, p<0.05); and hematocrit and hemoglobin were negatively correlated with IL-12 (r = −0.7 and −0.44, respectively, p<0.05).

**Fig 4:**
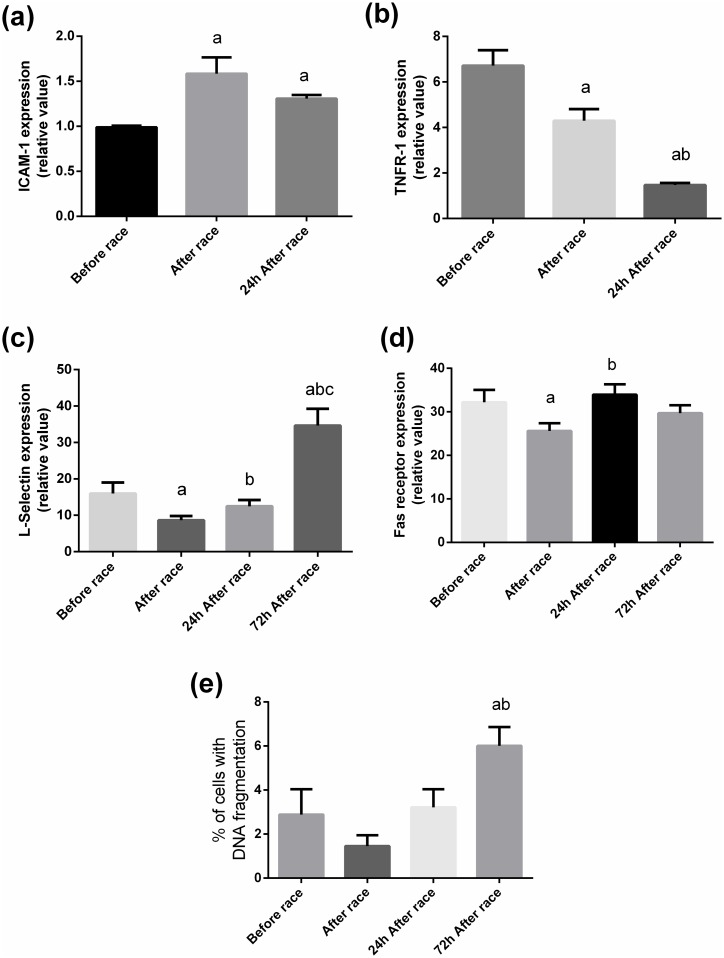
Effect of the marathon race on neutrophil surface molecules and DNA fragmentation. Neutrophils were separated after blood collection before, immediately after, 24 h after, and 72 h after the marathon race. Expression of ICAM-1 (A), TNFR1 receptor (B), L-selectin (C), and Fas receptor (D), and % of cells with DNA fragmentation (D) were determined. The fluorescence was determined by flow cytometry (BD Accuri cytometer). The values presented are the mean ± SEM of 21 runners. ^a^ p<0.05 vs before the marathon race, ^b^ p<0.05 vs immediately after the marathon race, and ^c^p<0.05 vs 24 h after the marathon race.

We observed an increased ICAM-1 immediately after and 24 h after the marathon race by 51% and 33%, respectively (Fig 4A), while L-selectin expression decreased immediately after race (from 15 ± 2 to 8 ± 1 relative value) and elevated 2- fold 72 h after competition (from 15 ± 2 to 34 ± 4 relative value) (Fig 4C). TNF receptor-1 (TNFR1) expression was decreased immediately after and 24 h after the marathon race by 40% and 80%, respectively (Fig 4B). Fas receptor expression reduced by 25% immediately after race and returned to baseline 24 h after marathon race. However, we observed an increased DNA fragmentation (by 2-fold) 72 h after marathon race (Fig 4D and 4E). Immediately after the marathon race, TNFR1 expression was positively correlated with L-selectin expression (r = 0.6, p<0.05) and negatively correlated with the percent of neutrophils with DNA fragmentation (r = −0.58, p<0.05). TNF-α levels were negatively correlated with L-selectin expression 24 h after the marathon race (r = −0.46, p<0.05).

## Discussion

Long-distance running induces muscle fiber damage and systemic inflammation followed by neutrophilia as previously demonstrated by other authors [3, 13, 19–22]. In this study, we also observed elevated levels of pro-inflammatory mediators such as IL-12, TNF-α, L-selectin expression in the recovery period (24 and 72 h after marathon), indicating a delayed neutrophil trafficking and death following exercise-induced muscular injury.

The incidence and severity of URTI was significantly greater after the marathon race. URTI correspond to 35 to 65% of illness presented in sports medicine clinic [2]. Identifying athletes at risk of recurrent URTI is important in order to prescribe preventative clinical, training and lifestyle strategies [1].

In this study, athlete’s performance, evaluated by V˙O_2peak_ and race duration, were associated with muscle damage markers. Exercise-induced muscle damage has an important impact on long-distance athletes performance and could be mediated by alterations in the sense of effort [23, 24].

**Fig 5:**
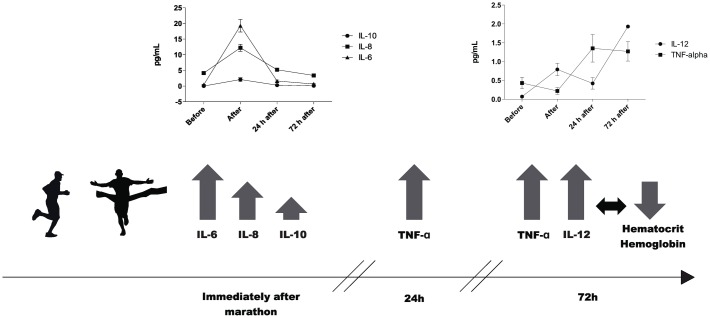
Model on the temporal dynamics of the cytokine’s responses to the marathon race.

The prevention and treatment to minimize and/or to accelerate muscle recovery after damage used target inflammation [25]. Muscle damage markers and IL-8, IL-6, IL-10, and IL-1ra plasma levels were increased after the marathon, and IL-6 appeared to remain elevated for up to 24 h afterwards. Some authors have been unable to detect changes in TNF-α, IL-12, IL-4, and IL-2 after a prolonged run, suggesting that IL-10, IL-1ra, and IL-6 may cause reduced pro-inflammatory cytokines to prevent systemic inflammation [3, 19, 26, 27]. However, in this study we observed an increased TNF-α and IL-12 plasma levels in the recovery period (24 and 72 h after the marathon race), and we suggested that these cytokine are induced by changes or balance between IL-10 and IL-6 levels (Fig 5). These controversial results could be explained because the most of studies evaluated TNF-alpha and IL-12 during, immediately or few hours after race and we observed from 24 hours after race [3, 19, 26, 27]. In an ultra-marathon study TNF-α fluctuated throughout the race with a significant increase at 308 km, approximately 64 hours after start the race [26]. Previous studies reported that plasma concentrations of IL-12 increased immediately after brief anaerobic maximal cycle ergometer exercise and in various intensities of treadmill running [28, 29]. IL-10 exerts an inhibitory effect on neutrophils function and may contribute to decrease on surface molecule expression (L-selectin, TNFR1 and Fas receptors). Previous study demonstrated relation between higher URTI and IL-10 production in response to antigen challenge in high levels of physical activity [30]. IL-6 is involved the activation of neutrophils and the coordination of the inflammatory response contributing to increase on ICAM-1 expression [4].

The hematocrit and protein reduction after race has been used to evaluate hemodilution after prolonged exercise and correct serum/plasma variables [31], but endurance exercise-induced hemolysis is a widely observed phenomenon as we demonstrated by 5% reduction on hematocrit/hemoglobin levels [32].

Kuvibidila et al. (2004)[33] suggested that iron deficiency associated with low hemoglobin and hematocrit alters cytokines, such as IL-12, affects innate immunity. In our results, we demonstrate a negative association between hematocrit/hemoglobin and IL-12 (Fig 5). IL-12 is an important pro-inflammatory cytokine that acts on innate and the adaptive immune systems by stimulating leukocytes, including natural killer cells of innate immunity to enhance cytotoxicity [34]. Recently study demonstrated that long distance exercise reduces the induction of in vivo immunity [35].

The increase in total neutrophil counts reflect a compensatory response to counteract the decrease in neutrophil functions [36]. Neutrophil extravasation is stimulated by inflammatory processes in an adhesion multistep cascade and transmigrated through the vessel wall, which required selectins for the rolling phase and adhesion molecules, such as ICAM-1 [16]. In an inflammatory environment, the velocity of neutrophils is dramatically reduced (slow rolling) leading to further neutrophil–endothelial cell interactions through the binding of leukocyte function-associated antigen-1 to its endothelial counter receptor ICAM-1 and triggering the neutrophil activation [16]. Bonsignore et al. (2001) [37] and Neilsen & Liberti (2004) [38] reported that few neutrophils in the induced sputum were positive for L-selectin after marathon in amateur runners. As a possible explanation, corticosteroid-induced down regulation L-selectin is supported by the correlation found between plasma cortisol and adhesion molecules [37]. Increased number of leukocytes, blood velocity, shear stress or catecholamine, excessive reactive oxygen species and growth hormone levels during exercise may also increase the risk level of endothelial dysfunction [39, 40]. In our study, we first observed an elevation on ICAM-1 expression (immediately after and 24 h after) and then on L-selectin expression (72 h after), indicating a possible slow rolling phase and a delay of neutrophil activation. Neilsen & Liberti (2004) [38] reported an elevation in the plasma levels of soluble of L-selectin after the marathon race that could contribute as a compensatory mechanism to high L-selectin expression in the recovery period. Jee & Jin (2012) [26] also demonstrated increased endothelial dysfunction markers, mainly sE-selectin, which was associated with leukocyte trafficking, exercise intensity, TNF-α, CRP, and CK. We also observed a correlation between changes on TNF-α and L-selectin 24 h after the marathon race, suggesting the importance of these cytokine on neutrophil trafficking in the recovery period.

The ability of pro-survival signaling to transiently delay apoptosis during neutrophil extravasation has been discussed [16, 17]. TNFα prolongs the neutrophil life span in low doses and induces apoptosis in high doses [17, 41]. In addition, the TNF-α in low doses after the marathon race was negatively correlated to the total number of leukocytes and neutrophils, and the high doses of this cytokine could be associated to neutrophil apoptosis in the recovery period. TNFR-1 and Fas are from the TNF-receptor superfamily classified into receptors carrying an intracellular death domain. In general, TNFR-1 triggers pro-inflammatory (anti-apoptotic) signals mediated by association of nielTNFR1 with TNFRSF1A-associated via death domain and receptor-interacting protein (RIP)1, and pro-apoptotic responses by recruiting another complex (complex 2 or death-induced signaling complex) explains contrasting results reported on the action of TNF-α on neutrophil apoptosis [15, 16]. Mooren et al. (2012)[11]proposed that different intensities of exercise induce a delay of neutrophil apoptosis that contributes to the maintenance of post-exercise neutrophilia and is followed by alterations of calcium, CRP levels, and redox status. In our study, the percentage of neutrophil with DNA fragmentation was negatively associated with TNFR1, and we suggested that the decreased expression of TNFR1 receptor and the increased Fas expression also contributed to the transient delay of neutrophil apoptosis.

In summary, the present data indicate that long-distance exercise affects neutrophil activation and apoptosis mediated by several factors such as TNF-α levels, L-selectin, TNFR1, and Fas expression. The half-life of neutrophils was modified by the course of inflammation induced by the marathon race and molecular pathways that control neutrophil survival holds therapeutic promise to reduce the susceptibility to infection in athletes. Therapeutic strategies, such as nutrition or cryotherapy, to modify the inflammatory mediator’s profile(TNF-α and IL-12 levels), mainly in the recovery period, could normalize the course of inflammation, neutrophil trafficking, and improve the runner’s immunity.
